# ‘Treatment Not Trident’: Medical Activism, Health Inequality and Anti-Militarism in 1980s Britain

**DOI:** 10.1093/shm/hky027

**Published:** 2018-04-19

**Authors:** Christoph Laucht

**Keywords:** medical activism, health inequality, anti-militarism, Medical Campaign Against Nuclear Weapons, Trident nuclear weapon system

## Abstract

In 1985, Britain’s chief group of medical anti-nuclear weapons activists, the Medical Campaign Against Nuclear Weapons (MCANW), launched its ‘Treatment, Not Trident’ (TNT) campaign. TNT called on the Thatcher Government to cancel the acquisition of the Trident nuclear weapon system and divert those funds to the National Health Service and foreign aid instead. Using TNT, this article makes some more general observations about key aspects of the history, nature and ideologies of medical activism in relation to anti-militarism and health inequality. Alongside a conceptualisation of ‘medical activism’, it offers an examination of chief ways in which the strategic mobilisation of health and welfare priorities, and a growing interest in developing nations enabled MCANW to reach a larger audience. Moreover, higher levels of professionalisation, politicisation and inclusivity contributed to TNT’s success, making it a crucial moment in the development of both MCANW and medical activism in general.

Upon his arrival at the Conservative Party conference in Manchester in October 2015, the Secretary of State for Health Jeremy Hunt faced loud protests over his plans to reform the National Health Service (NHS), which represents both an integral component and symbol of the British welfare state. One of the placards on display outside the conference venue boasted one of the slogans of the Campaign for Nuclear Disarmament (CND)—‘NHS Not Trident’.^1^ This motto linked the funding crisis of the NHS with the pledge of the Cameron Government to renew the Trident nuclear weapon system, which comprised American-made submarine-launched Trident ballistic missiles (SLBMs) armed with British-manufactured nuclear warheads and based on British-built nuclear submarines.^2^ But such criticism of the proportionality of spending on defence versus public health was hardly new, for it went to the heart of an anti-militarist argument that medical activists such as the Prussian pathologist, politician and peace campaigner Rudolf Virchow had promoted as early as the second half of the nineteenth century.^3^ While CND used a similar rhetoric in its original 1980s campaign against the Thatcher Government’s decision to purchase Trident and compared, amongst other things, government expenditure on that weapon system to ‘the cost of 500 general hospitals’, the Medical Campaign Against Nuclear Weapons (MCANW) targeted the imbalance between the government’s overspending on nuclear arms and its simultaneous underfunding of the health services and foreign aid in a separate ‘Treatment Not Trident’ (TNT) campaign.^4^ In TNT, which marked MCANW’s first national campaign, Britain’s chief group of medical anti-nuclear weapons activists called on the Thatcher Government to cancel the procurement of the Trident system and re-allocate those funds to the NHS and foreign aid budgets instead.^5^

This article uses TNT to make some more general observations about key facets of the history, nature and ideologies of medical activism in relation to anti-militarism and health inequality in 1980s Britain and beyond.^6^ Alongside a conceptualisation of ‘medical activism’, it offers an examination of key aspects of this phenomenon. In particular, it explores the strategic mobilisation of health and welfare priorities, and a growing interest in developing nations. Through the combination of health inequality with nuclear disarmament—one of MCANW’s original objectives—TNT addressed a matter that directly affected many Britons, thus making the campaign relevant to a larger audience. Similarly, a broader geographical focus on the so-called Third World spoke to contemporary concerns past the nuclear weapons issue. What further boosted the campaign’s impact was MCANW’s readiness to formulate more pronounced political statements in the context of TNT than in previous declarations, the increasing professionalisation of its campaigning methods and style and the intention to raise its level of inclusivity by recruiting more health workers, especially nurses.^7^ By addressing these issues, this study brings medical activism into conversation with the historiographies of non-governmental organisations (NGOs), especially their evolution from single- into multi-issue campaigns during the 1980s, the welfare state, Thatcherism and anti-nuclear weapons protests.^8^

Central to MCANW’s campaigning against nuclear weapons was a notion of ‘medical activism’ that drew legitimisation from the occupational backgrounds of the group’s members, in particular their commitment to specific professional codes of ethics and expertise. This dated back to 1980, when concerned medical professionals, at a time of rising tensions between the superpowers, launched MCANW as a group of medical and health experts under the directorship of immunologist John Humphrey to examine the anticipated medical consequences of nuclear war.^9^ The group’s reliance on ‘contributory expertise’, or the highest level of know-how, about the medical effects of nuclear war distinguished its work from public health activism such as the AIDS movement with its heterogeneous lay supporter base that commonly relied on ‘interactional expertise’ and lacked a more profound medical understanding of the subject matter.^10^ Yet, with its relatively small membership base of about 3,500 and its fairly centralised organisational structure, MCANW also shared key characteristics with public health groups and, as Virginia Berridge and Alex Mold observe elsewhere, ‘drew strength from an image, rather than a reality, of mass activism’.^11^ This self-fashioned contributory expert identity also fostered a high degree of exclusivity amongst MCANW’s membership, with medical professionals (63.4 per cent) outnumbering nurses and other health workers by far.^12^ And this was despite the fact that the Nursing Campaign Against Nuclear Weapons had merged with MCANW in 1982, and MCANW had subsequently set up a Nurses Working Party.^13^

Apart from its contributory expert identity, MCANW’s reluctance to take political positions prior to TNT limited its impact. Like other British NGOs, MCANW campaigned from a platform situated in-between pressure groups like the Abortion Law Reform Association or Lesbians and Gays Support the Miners, on one side, and social movements such as CND or political campaigning organisations like the Socialist Medical or Socialist Health Associations respectively, on the other.^14^ Since many medical professionals subscribed to an ideology of medicine being a ‘depoliticised profession’ and MCANW was, in principle, open to all medical professionals and health workers regardless of their political persuasion, the group was cautious not to establish close links with CND; for such a move might have alienated members. Even though MCANW comprised many members with centre-left political allegiances, the group remained, in the words of Steve Watkins, ‘a broad-based campaign of ordinary doctors concerned at the drift into nuclear war’ that lacked experience in medical politics.^15^

MCANW’s participation in transnational networks also influenced its agenda and transformation into a multi-issue campaign. Alongside the Medical Association for the Prevention of War (MAPW), MCANW became one of the two British affiliates of the International Physicians for the Prevention of Nuclear War (IPPNW)—the main transnational hub of medical anti-nuclear weapons activists during the 1980s and winner of the 1985 Nobel Peace Prize.^16^ MCANW, like other IPPNW affiliates, adopted a prophylactic medical approach to the nuclear arms race and, in conjunction with IPPNW, frequently issued ‘medical prescriptions’ on nuclear-arms-related matters to reduce the risk of nuclear war. While this preventative approach represented a general underlying ideological pillar of MCANW’s medical activism, it was IPPNW’s diagnosis of militarism, in connection with schizophrenia, as ‘a psychosocial disease’ and of militarists as ‘patient[s]’ or ‘[v]ictims of militarism’ that informed TNT’s anti-militarist message.^17^ After all, Trident exemplified a particularly powerful, technology-driven form of what David Edgerton terms ‘liberal militarism’ within Britain’s ‘warfare state’.^18^ In this, MCANW’s and IPPNW’s diagnosis echoed views expressed by psychiatrists and the Medical Peace Campaign as well as the Czechoslovakian paediatrician Emil Flusser in his pioneering book *Krieg als Krankheit* (*War as Illness*) during the 1930s.^19^

If international issues framed many of MCANW’s aims and objectives, the group operated simultaneously within a particular national context and addressed genuinely British issues. Unlike other Western European nations such as West Germany or Belgium, Britain possessed an independent nuclear deterrent. At the same time, TNT addressed problems that were peculiar to the British health services. Consequently, MCANW’s anti-nuclear weapons activism developed dynamics and trajectories that differed from IPPNW affiliates in other countries.

The present study progresses in two stages. Its main section examines the evolution of MCANW and its medical activism in TNT. Particular emphasis is placed on the group’s programmatic expansion beyond a single-issue, anti-nuclear weapons campaign through the combination of MCANW’s traditional campaigning focus on nuclear disarmament with questions of health inequality and global development as well as its growing politicisation, professionalisation of its campaigning methods and inclusive approach towards nurses and other health workers. A second section then explores the ways in which TNT inspired subsequent MCANW campaigns, demonstrating the ongoing diversification of the group’s agenda. Throughout, this article investigates MCANW’s anti-nuclear weapons activism within both the synchronic and diachronic contexts of medical and peace campaigning as well as NGO work.

## ‘Treatment Not Trident’, 1985–86

MCANW officially launched TNT with a press conference in the House of Commons on 23 September 1985. Hosted by the shadow Minister for Health Frank Dobson (Labour), the event featured such illustrious speakers as paediatrician Helen Caldicott, a founding member of MCANW and former president of its American sister organisation Physicians for Social Responsibility, and Sir Douglas Black, who had previously served as president of both the Royal College of Physicians and the British Medical Association (BMA) as well as Chief Scientist at the Department of Health and Social Security (DHSS).^20^ Additional events took place in Aberdeen, Birmingham and Sheffield, with Caldicott delivering further talks in Edinburgh and Glasgow.^21^ To lend TNT authority, MCANW secured prominent support from biophysicist Maurice Wilkins, medical physicists Jack Boag and Joseph Rotblat, dermatologist Sam Shuster, haematologist Allan Jacobs, Labour MP Robin Cook and the leader of the Liberal Party David Steel. The fact that Brigadier Michael Harbottle and Air Commodore Alastair Mackie, two ‘unorthodox defencists’, as Martin Ceadel calls such military figures who opposed Trident in favour of higher expenditure on conventional military forces, also backed the campaign further strengthened TNT’s argument.^22^

MCANW placed the imbalance between excessive government expenditure on a nuclear weapon system and chronic underfunding of the health services and foreign aid at the centre of TNT. It proposed a pragmatic solution: the Thatcher Government should scrap the Trident programme and re-invest the funds thus saved into the NHS and aid for developing nations. In this, MCANW combined nuclear disarmament, one of its traditional key objectives, with a new focus on health inequality in Britain and developing nations. This act of unilateral British nuclear disarmament represented, in MCANW’s official view, the first stage in a process aimed at achieving multilateral nuclear disarmament (particularly a comprehensive nuclear test ban) and ultimately a global ‘freeze’ on nuclear arms development and manufacture.^23^

By making unilateral British nuclear disarmament part of a broader multilateralist argument, MCANW sought to defuse the highly politicised and unpopular issue of unilateralism. After all, the Labour Party, which pledged to abolish the British nuclear deterrent, suffered a humiliating defeat in the 1983 general election.^24^ While unilateral nuclear disarmament was never a popular proposition in Britain, growing public doubt over the high expenditure on the Trident system appeared to provide MCANW with an opportunity to rally public support behind TNT. By December 1984, the Thatcher Government feared that the opposition to Trident by the Labour Party and the Social Democratic Party–Liberal Alliance might play into the hands of CND.^25^ MCANW exploited this mood by citing another ‘unorthodox defencist’, Field Marshal Lord Carver, who referred to Trident as ‘a waste of money’, in campaigning materials.^26^

MCANW’s line on nuclear disarmament closely followed policy decisions by IPPNW and the BMA, the chief representative body and union of British medical professionals.^27^ Although MCANW decided in 1983, in line with a recent BMA resolution, not to promote unilateral nuclear disarmament because a unilateralist line might alienate some members, the group subsequently connected unilateral and multilateral nuclear disarmament through its simultaneous support of an IPPNW motion that called on world governments to instate a multilateral ‘freeze’ on the manufacture, siting and testing of nuclear arms as a first step towards the eventual elimination of all nuclear weapons. As part of this multilateral ‘freeze’, MCANW urged the Thatcher Government to abandon its plans to procure the Trident system and demanded that American cruise missiles were not deployed to Britain.^28^ The decision by the BMA’s annual representative meeting in July 1984 to endorse multilateral global disarmament—both nuclear and conventional—including a call on world governments to re-allocate defence funds to health care programmes at home and in developing nations appeared to legitimise both MCANW’s evolution into a multi-issue campaign and TNT’s anti-militarist argument further.^29^

**Figure 1:**
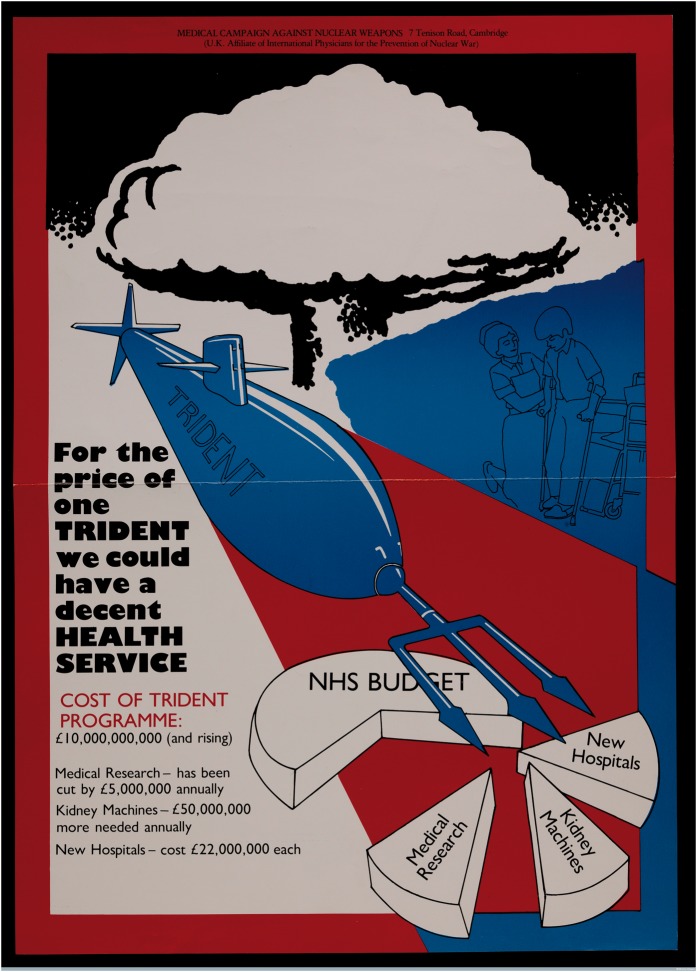
‘Treatment Not Trident’ campaign poster. Item from the Medact Archive. Wellcome Images reference: L0075381.

The campaign poster visualised TNT’s anti-militarist message about health inequality, depicting a Trident nuclear submarine (with a trident mounted on its bow) and a pie chart that symbolised the NHS budget being torn apart in three areas (medical research, new hospitals and kidney machines) by the submarine’s vicious-looking trident (Figure 1). Funds set free through a cancellation of the Trident purchase, argued MCANW, could be used to invest in these chronically underfunded, yet crucially important areas of the health care sector. By comparison with the multi-billion expenditure on the new nuclear weapon system, the Thatcher Government cut the budget for medical research by £5 million annually—a comparatively small yet, from MCANW’s point of view, vital sum. Similarly, MCANW assessed the cost of crucially needed kidney machines to be in the region of £50 million per year and the cost for one new hospital to be £22 million. Through these comparisons MCANW sought to demonstrate the levels of improvement that a cancellation and re-allocation of the Trident budget to the NHS might in theory support.

Two publications accompanied TNT, outlining its main aims and arguments. The first one was a short pamphlet entitled *Trident or Health?* It offered a brief technical overview of the Trident system and emphasised the technological dependence of its American-made missiles on the United States military and NATO policy.^30^ The second publication took the form of a more substantial booklet, entitled *A Tragedy for the Health Services*. Drafted by the TNT Working Group, which MCANW set up for this purpose, the national office distributed the report. Unlike the shorter TNT pamphlet, it contained references throughout, thus suggesting to readers a high degree of academic inter-subjectivity and medical expertise.^31^ In addition, MCANW intended to table an early day motion, a common approach taken by NGOs, on government expenditure on health versus Trident in the House of Commons.^32^

TNT, thus, marked a decisive moment in MCANW’s transformation process from a single- into a multi-issue campaign: although Trident ‘surfaced’ on MCANW’s agenda from November 1982, it did not take centre stage in the group’s campaigning until December 1984, when MCANW’s Executive Committee acknowledged the need for the group’s programmatic diversification. In light of declining public and media interest in MCANW’s traditional focus on the medical effects of nuclear war, the board decided that the imbalance in government spending on Trident and the NHS, in particular the resultant health inequality, should become its central campaigning focus.^33^ The timing of the Executive Committee’s decision coincided with the final period of the Second Cold War when tensions between the superpowers and public fears of nuclear war abated.^34^

This programmatic shift mirrored developments within the wider NGO sector and beyond the anti-nuclear weapons movement. After the Second World War, the increasing secularisation and professionalisation of British society replaced religious beliefs with professional expertise and codes of ethics as the underlying principles of NGO work. For many NGOs, this trend initially translated into an orientation as single-issue campaigns, based on their members’ professional expertise and ethos.^35^ During the nuclear test ban debate of the 1950s, for example, atomic scientists played a crucial part in informing the public and political decision makers about the human health and environmental effects of nuclear weapons.^36^ By the mid-1980s, however, MCANW, like OXFAM and other NGOs, deemed it vital to adopt a multi-issue agenda to ensure its continued relevance and, ultimately, its existence.^37^

MCANW’s transformation into a multi-issue campaign was entwined with its growing readiness to take political positions. ‘Patients will be given a little preventive medicine’, alluded MCANW chairperson Stephen Farrow at the TNT press conference to one key target group for TNT and justified its political message—a chief characteristic of that campaign. National newspapers and medical journals also registered TNT’s comparatively high degree of politicisation. And TNT marked, according to the *Guardian*’s medical correspondent Andrew Veitch, ‘the first time that a large chunk of the medical profession in Britain ha[d] taken political action on nuclear weapons’.^38^

This politicised tone found expression in TNT’s anti-militarist rhetoric, especially comparisons revolving around the cost of the nuclear weapon system. An MCANW press communiqué equated Trident’s estimated cost of £11 billion to the sum of ‘£30,000 per day for the next thousand years’.^39^ MCANW relied on existing arguments against Trident that had emerged in the context of the public debate over its cost and strategic relevance around the time of the first Trident deal.^40^ When the Thatcher Government was forced to revise its order of Trident missiles from the C-4 to the much more powerful D-5 model because of decisions taken by the incoming Reagan Administration in the United States, resistance against that weapon system grew from both within and outside the Conservative Party.^41^

Given the great uncertainty about the definitive cost of Trident, MCANW put different price tags on that weapon system at different stages of TNT, ranging from about £9 to £11 billion.^42^ This appeared to be in line with official and unofficial estimates of the cost of procuring the D-5 model that varied between some £7.5 billion and £33.1 billion for the period from 1981 to 1992, depending on a complex set of factors such as the exchange rate between the British pound sterling and the United States dollar or the cost of refitting the missiles for their British-designed carrier submarines.^43^

To back up its anti-militarist line, TNT provided statistical evidence of disparities in government funding for defence and health care. *Trident: A Tragedy for the Health Services* used data collected by the National Association of Health Authorities that showed a rise in expenditure on the NHS by 0.59 per cent in real terms in the fiscal years from 1978–79 to 1983–84, while the defence budget had grown by about 3.91 per cent per annum during the same period. Internationally, MCANW argued, Britain, which spent 5.4 per cent of its gross national product on the health services in 1984, trailed behind other Western nations, including Sweden (10 per cent), the United States (9.5 per cent), West Germany (8 per cent) and France (6 per cent).^44^ This international comparison was misleading though, as it did not factor in the cost effectiveness of individual health care systems, especially the American one that had just experienced a drive towards commercialisation and seen the rise of a ‘new medical-industrial complex’ at the expense of affordable patient services.^45^

Besides relying on budgetary data, MCANW established a correlation between infant mortality rates and the prioritisation of government spending on defence over health care to accentuate the detrimental impact that such disproportionate government spending on the military could have on health care provision. Since infant mortality represented a chief marker of health inequity, this causal link offered a powerful evidence-based tool to support MCANW’s anti-militarist argument. TNT materials cited two historical examples that had previously been used at IPPNW’s 1984 international congress as evidence of such a direct connection. In the United States, the group claimed, an increase in the defence budget led to welfare cuts between 1980 and 1982. As a result, the child mortality rate remained at a relatively high level for an industrialised nation. By contrast, infant mortality rates in Costa Rica dropped sharply after the country had stood down its military forces in 1949, thus setting a positive example for MCANW’s anti-militarist line in TNT. Although perinatal mortality rates in Britain had been in decline for years, the group noted with concern that those for infant mortality remained at a relatively high level by comparison with other Western countries such as Sweden, Japan, France or Spain. And, what is more, MCANW even took into account socio-demographic factors: ‘For the death of every one male infant born to professional parents, almost two will die among children of skilled manual workers and three among the children of unskilled manual workers.’^46^

Two studies informed TNT’s line of argumentation on the causal relationship between militarism, health inequality and infant mortality. The first one was a report by two American physicians, Steffie Woolhandler and David U. Himmelstein, on the link between infant mortality, health inequality and military expenditure. It was based on data from 140 nations and concluded ‘that militarism [wa]s deleterious to health even in the absence of overt hostilities’ and would result in ‘two million infant deaths each year’.^47^ The second one was the so-called Black Report on health inequality in Britain. Its publication in August 1980 signified a key moment for re-focusing the attention of medical professionals and health workers on health inequality as a major issue: while it represented a chief concern in the aftermath of the Boer War and during the 1930s global economic crisis, health inequality only re-surfaced as a key matter for medical professionals and health workers in the 1970s.^48^

Officially called *Inequalities in Health*, the Black Report was the product of a Working Group on Inequalities in Health in the DHSS under the directorship of Sir Douglas Black. It established a correlation between poor health and low socioeconomic status that supposedly affected members of low-income groups throughout their lives. Its authors attributed this link to causes outside the remit of the NHS such as education, income or unemployment and argued for a radical overhaul of health policy and the NHS as well as providing more aid to lower-income groups.^49^ With these demands, the Black Report and TNT added their voices to concerns that had been expressed by medical professionals since the time of the public health movement in Victorian Britain.^50^

The ignorance and indifference that the Thatcher Government displayed towards *Inequalities in Health* caused consternation and anger amongst medical professionals, including Black’s team and MCANW. *Trident: A Tragedy for the Health Service* cited a statement by the Secretary of State for Health Patrick Jenkin, in which the latter dismissed calls by Black’s team for ‘additional expenditure’ in the region ‘upwards of £11 billion a year’, the equivalent to the estimated cost of the Trident system, as ‘quite unrealistic in present or any foreseeable economic circumstances’, as proof of the Thatcher Government’s prioritisation of Britain’s nuclear deterrent over public health care.^51^ What further added to this impression was the way in which the government handled the missive by comparison with its open endorsement of the purchase of the Trident system. Contrary to established practice, the DHSS neither issued a press release nor held a press conference, and neither Her Majesty’s Stationary Office nor the DHSS printed it. Instead, the DHSS distributed photocopies of the report to select journalists on the Friday before the 1980 August Bank Holiday weekend.^52^ By contrast, the government openly promoted the decision to acquire Trident missiles through a reprinted exchange of letters between the Thatcher Government and the Carter Administration on the first Trident deal in *The Times*.^53^

If the budgetary prioritisation of nuclear armaments over public health care put the health services in a financially difficult position, the simultaneous ‘“Thatcherization” of the NHS’ (Martin Gorsky) placed them under even greater pressure, thereby creating another major motivation behind TNT. Apart from the comparatively low growth rate of real expenditure on the health services (the lowest in about 30 years), the increasing bureaucratisation, a higher level of engagement with the private sector, the introduction of rigorous managerialism and the appointment of an NHS chief executive in the wake of the 1983 Griffiths Management Inquiry worried many medical professionals and health workers.^54^ While the ‘welfare state did in fact achieve substantial reductions in absolute (but not relative) inequalities in overall health and income’ between 1945 and 1980, as Simon Szreter argues, it eventually became a victim of its own success. And its accomplishments in creating greater social security and affluence, particularly amongst members of the working classes, ironically prepared the ground for promoting the Thatcherite ideology of ‘“opting out” of the expensive measures of collective state provision’.^55^

Alongside reforms within the NHS, the perceived state of crisis that the health services faced at the time also made the protection of the welfare state, from MCANW’s perspective, a highly relevant campaigning item.^56^ In TNT, MCANW thus offered a diagnosis of the state of the NHS. The group set out to debunk claims by Norman Fowler, Jenkin’s successor as Secretary of State for Social Services, that government expenditure on the NHS had increased by 17 per cent in the period from 1978 to 1984 on the grounds of three ‘complicating factors’ that Fowler had failed to take into account. Since price increases for services and goods continuously floated above the inflation rate, the group argued that this increase constituted in fact only 7 per cent in real terms. The second factor concerned the fixed nature of the overall budget for the NHS, with a rise in funding in one area of the health services necessitating cutbacks in another. To illustrate this, MCANW pointed to recent, additional investment in the health services in northern England that prompted in turn budget cuts and hospital closures in the London area. Finally, the group argued that continuing technological and demographic developments alone required an increase in the NHS budget by 1.5 per cent per annum for the health service to be able to operate at a steady level.^57^

Besides general funding, TNT identified several areas where health care provision had suffered severely over the past few years, particularly a net reduction in the number of hospital beds by about 12,900 between 1980 and 1983. Here, MCANW cited a study by the Radical Statistics Group, an activist group of statisticians, on health inequality. During the winter of 1984–85, these cuts in bed numbers caused a crisis in London, preventing many general practitioners from admitting patients to hospitals and resulting in many fatalities. Another area concerned staffing in the NHS. In spite of an increase in the number of nurses, the group argued that simultaneous cuts in numbers of auxiliary nursing staff and a rise in workloads had in fact eliminated any growth in staff numbers. What further hampered improvement in the quality of public health care, in MCANW’s view, was the Thatcher Government’s refusal to create better consultant–junior doctor ratios and career opportunities for junior staff. If these issues were grave, the report also flagged up waiting lists for out-patient and non-urgent hospital treatments as chief indicators of the underfunded status of the NHS and the resultant inability to meet its demands. ‘These [we]re particularly marked for many disabling and often painful conditions including blindness, deafness and arthritis which [we]re not considered to pose an urgent threat to life’, stressed *Trident: A Tragedy for the Health Services*. To put this point into perspective, the booklet referred to a continual increase in patient numbers on waiting lists from about 500,000 in 1975 to some 700,000 in 1983 in spite of protests by medical practitioners and changes to the data collection that excluded day procedures from 1979.^58^

Together with its diagnosis of the state of the NHS, and in line with its preventative medical approach, MCANW singled out six areas where strategic investment of funds saved by abandoning Trident could effectively be used to improve conditions in the health services. Apart from general practitioners in urban, especially inner city, areas with high levels of unemployment, poverty and disadvantaged social groups, TNT campaigning materials pointed out that care for the elderly was in desperate need of increased funding. With population ageing likely to prompt major changes in Britain’s demographic make-up over the next decade or so, the report stressed that these developments might necessitate, for example, a 20 per cent increase in hospital beds alone over the next 10 years. Moreover, it viewed screening for cervical cancer as an important, yet severely underfunded area within the NHS. The same applied to treatments of chronic renal failure on which the government spent some £60 million annually, which amounted to ‘less than the cost of a single Trident missile’. Other fields in need of more funding were NHS services dealing with drug addiction and medical research, especially the Medical Research Council.^59^

While the campaigning angle on underfunded areas within the British health services demonstrated MCANW’s broader programmatic remit as part of its evolution into a multi-issue campaign, TNT also comprised a wider geographic focus on developing nations. This shift in MCANW’s agenda reflected a growing public awareness of health crises in developing nations. In 1981, the Overseas Development Administration, the government department in charge of foreign aid, received numerous complaints by members of the public who protested against cuts to the foreign aid budget, often suggesting a re-allocation of funds from the defence to the foreign aid budget.^60^ As for MCANW and TNT, IPPNW’s Fourth World Congress in 1984, which was attended by representatives from MCANW and other national affiliates, laid some important groundwork by addressing questions of military spending in relation to public health, with special reference to developing nations.^61^ But it was the Ethiopian famine of 1984–85 that provided an immediate context for TNT; for it sensitised many Britons and people around the world to the plight of Ethiopia and prompted a major humanitarian response through the Band Aid and Live Aid projects or NGOs such as Médecins sans Frontières and the British Disasters Emergency Committee, which comprised the British Red Cross, the Catholic Fund for Overseas Development, Christian Aid, Oxfam and Save the Children.^62^

TNT addressed ‘world health needs’ in a separate pamphlet in stark political language (‘The cost of a 20 year programme to provide essential health and food needs for all Third World countries is less than the worldwide yearly budget for nuclear weapons.’). The leaflet alerted readers to the perils that malnutrition and infectious diseases, in particular malaria, trachoma and bilharzia, posed to people in the ‘Third World’. It even claimed that some ‘150 people w[ould] have died needlessly in the poorer countries of our world’ during the time required to read the pamphlet and suggested that, for the majority of mankind, the concept of global health represented nothing more than ‘a cruel joke’. In a similar vein, the leaflet connected MCANW’s original remit of nuclear disarmament with its new focus on developing nations by equating ‘[t]he number of children dying of hunger and poverty … to the dropping of the Hiroshima bomb somewhere in the Third world every two or three days’. MCANW regarded the inequalities that many people in developing nations faced to be a direct result of Western progress since the days of nineteenth-century European colonialism. And both nuclear and conventional armaments now exacerbated these inequalities.^63^

Like MAPW and other NGOs, MCANW cited the findings of the Brandt Report as justification for the urgent need to discuss ‘Third World’ development issues.^64^ The missive was named after the former West German chancellor Willy Brandt, who directed the North–South Commission that investigated the social and economic conditions in developing nations for the World Bank with support from the United Nations. It called on Western countries to assist developing nations with the improvement of their agricultural production, the provision of both clean water and essential health care to their populations as well as the elimination of diseases such as malaria and bilharzia.^65^

In line with Brandt’s proposals, TNT urged the Thatcher Government to increase its foreign aid budget from 0.35 per cent of the country’s gross national product to the levels recommended by either the United Nations (0.7 per cent) or the Brandt Report (1 per cent). Simultaneously, MCANW stressed the need for implementing nuclear and conventional disarmament to set free funds that could then be used for providing inexpensive yet effective measures to reduce infant mortality in developing nations. Amongst these was the provision of growth charts to monitor children for early signs of malnutrition, large-scale immunisation against tuberculosis, yellow fever and measles as well as diarrhoea treatment. Furthermore, TNT materials used the cost of a single fighter aircraft to illustrate how those 20 million US dollars could alternatively be used to pay for some ‘40,000 village pharmacies’.^66^

Finally, an increasing professionalisation of campaigning methods and styles accompanied MCANW’s evolution into a multi-issue campaign. Like other NGOs of the public health and foreign aid sectors, MCANW optimised its use of campaigning materials and interaction with the news media.^67^ Here, the group also sought advice from OXFAM.^68^ The creation of a Strategy Group in charge of identifying, planning and coordinating campaigns, with John Launer at the helm, in the lead-up to TNT, marked one of the chief steps in advancing the professionalisation of MCANW’s campaigning techniques.^69^ Besides targeting the news media and Members of Parliament (MPs) to generate publicity, some of the group’s chief actions constituted the establishment of more effective communication with local branches to promote the campaign at the grass-roots level and the re-wording of the campaign focus ‘from “cuts” to “needs”’ to convey a more positive and readily documented message.^70^

Alongside mobilisation of members both at branch and national levels, these efforts comprised collaboration with the Joint Parliamentary Committee, which the group operated with MAPW, as well as the production of some of the first campaign materials.^71^ To prime members at the grass-roots level on effective ways of promoting the campaign, a set of notes emphasised the importance of reaching out beyond medical professionals to health workers. In addition, they offered practical guidance on how to liaise with other relevant groups, raise funds, optimise internal communication, lobby local MPs, plan public events effectively and make efficient use of key arguments against Trident. ‘Television companies do not work at weekends or in the evenings unless you are Neil Kinnock or Margaret Thatcher; so plan events inside working hours if you want to try for coverage’, press officer Su Maddock instructed MCANW members pragmatically in these materials.^72^ Apart from a second information pack, the Strategy Group supplied local branches with a resources pack that contained, amongst other things, a TNT leaflet, car sticker and pin badge, a list of relevant BMA and MCANW resolutions, sample publicity materials to show branches how they could produce campaigning materials relating to their local areas and a sample letter to a local MP.^73^ And, as this preparation demonstrates, MCANW had not only developed into a multi-issue campaign that issued more pronounced political statements, but the group had also undergone a significant professionalisation process in its campaigning styles and methodology.

## From ‘Treatment Not Trident’ to ‘Beds Not Bombs’ and the ‘Third World’

Almost immediately after the TNT launch, MCANW began with its assessment of and reflection on that campaign, including plans for taking it further. The Strategy Group was satisfied with the launch event, particularly the ‘good press and media coverage’.^74^ The latter drew considerable interest from general practitioners and hospital doctors.^75^ Its popularity, together with the practice of distributing leaflets to patients via GPs and photocopying materials, forced the Executive Committee, by October 1985, to provide additional funds for the production of extra leaflets to meet the demand.^76^

Although MCANW’s new political mindedness made TNT more appealing to a public audience, it prompted mixed responses from professional bodies, the clergy, anti-nuclear weapons activists and local health authorities. While the BMA recognised ‘the need for better funding in the National Health Service’, it did not actively support TNT in line with its strict opposition to unilateralism. The Royal College of Nursing was more amenable to the campaign, promoting it amongst its members. The British Council of Churches, in principle, supported TNT, and the Bath Anti-Trident Action Group even ordered copies of the campaign booklet.^77^ Local health authorities where many MCANW members were employed did not always look too kindly to TNT’s overtly political message. Therefore, some members’ employment status prevented them from promoting TNT in hospitals, with some nurses even losing their jobs as a consequence of their open support of TNT.^78^

Nevertheless, MCANW decided to take TNT forward. And, from November 1985, further plans started to shape up. Besides seizing the opportunity of using the award of the Nobel Peace Prize to IPPNW in December 1985 as ‘a vehicle for publicising’ TNT, MCANW sought to increase involvement at grass-roots level. In addition, the group envisaged a stronger engagement with nurses to strengthen the role of health workers within its ranks.^79^ Although by the end of its first year, the recruitment rate of new members had quadrupled, with proportionally more health workers than medical professionals now joining MCANW, medical professionals still dominated the group.^80^ MCANW, thus, targeted all London nurses with a leaflet campaign to recruit more health workers. These efforts came to fruition with a nurses’ TNT bus tour through England in May and June 1986.^81^

**Figure 2:**
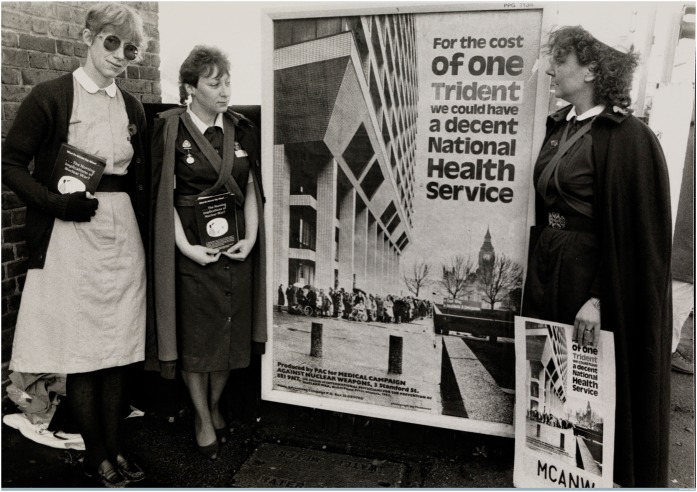
Nurses with ‘Treatment Not Trident’ re-launch campaign poster. Photograph from the Medact Archive. Wellcome Images reference: L0075324.

By early 1986, plans for a continuation of TNT and a successor campaign were taking on more concrete shape. The Strategy Group called on branches to support the campaign by exploring possible links with health trade unions and the Labour Action for Peace. In addition, the team produced a new TNT campaign poster that was later on display at over 60 sites across Britain. It showed patients queuing outside a hospital, along with the anti-militarist line ‘For the cost of one Trident we could have a decent National Health Service’ (Figure 2). While the Strategy Group envisaged the re-launched TNT campaign to have a wider focus on global health issues, MCANW was also set to start a new national campaign on the connections between health in developing nations and defence spending in 1987.^82^

MCANW deliberately chose 23 September 1986 as the launch date of the TNT update to mark the first anniversary of the original campaign start.^83^ A press release explained the rationale behind MCANW’s decision to continue with TNT in modified form. Since the launch of the original campaign a year earlier, it cautioned, ‘the cost of Trident ha[d] risen by almost £600 million and the keel of the first submarine ha[d] already been laid by Mrs Thatcher’. However, the NHS had faced ‘a shortfall of more than £6 million for every health district in England and Wales’ during the same period—an assessment that the *British Medical Journal* shared.^84^

The TNT update raised, by and large, similar issues as the original campaign and was also aimed at increasing membership from health workers in the lead-up to an entirely new national campaign to be launched in 1987.^85^ Yet, one major difference concerned the emphasis that the update placed on unemployment as a chief root of infirmity and poverty. This new angle was a response to the high unemployment figures that had remained above three million between 1982 and 1986, shifting public attention at times away from nuclear arms.^86^ Here, TNT followed in the tradition of sociological and psychological studies of the consequences of unemployment that had emerged during the 1930s world economic crisis.^87^ Curiously though, in the wake of the Chernobyl reactor accident in the Soviet Union in April 1986, civilian nuclear energy did not feature predominantly in MCANW’s campaigning, let alone TNT.^88^

At the same time, MCANW’s message and rhetoric advanced even deeper into political territory. A press release condemned ‘a lack of political will and investment’ for the absence of ‘fairness in the health field’, while the Thatcher Government spent vast sums on Trident—‘a new and destructive weapon of genocide’.^89^ With its reference to nuclear arms as genocidal weapons, TNT touched on emerging discourses over human rights and the illegality of nuclear weaponry. While Oxfam started to apply a human rights discourse to questions of development in the ‘Third World’, the contributory expert group Lawyers for Nuclear Disarmament called for nuclear arms to be outlawed.^90^ In this context, MCANW labelled the decision to purchase Trident ‘irrational and immoral’ and warned that the government’s funding priorities might create a scenario where ‘our scale of values [wa]s no longer worth defending’.^91^ And this marked a strong politicisation—not only by MCANW’s standards.

The accompanying booklet echoed this political tone and offered, in the words of MCANW’s new press officer Gillian Reeve, ‘a chilling update’.^92^ With 25 pages, it was almost three times as long as *Trident: A Tragedy for the Health Services*, thus giving MCANW considerably more space to explain its content and argument. The new booklet was referenced throughout and contained an additional section on Trident in relation to several (often quite political) issues. Besides moral-ethical quandaries about Trident’s legitimacy (‘an insult to morality’) and consideration of its role in international security and relations in terms of ‘an obstruction to the progress of arms control’, the booklet shed critical light on technical features of Trident, in particular its alleged technological dependence on the United States military.^93^

The great uncertainty surrounding the exact cost of that weapon system remained a source of concern for MCANW. After all, the Trident decision had already resulted in a dramatic increase in Britain’s defence budget to £18.2 billion (a higher proportion of the national income than in any other NATO member state except for the United States) compared to £16.7 billion spent on health care. In addition, the report questioned the Thatcher Government’s claim that the acquisition of the Trident system would create more jobs, especially in shipyards in Barrow-in-Furness, Cumbria, that built the Vanguard class carrier submarines. MCANW estimated that approximately 50 per cent of Trident-related jobs would be created in the United States. Given the high cost of that weapon system, MCANW—like many anti-nuclear weapons campaigners—followed a ‘defencist’ line, arguing that Trident ‘pushed out conventional projects’ in the British defence industry and hence jeopardised jobs.^94^

Finally, the TNT update criticised the secretive and un-democratic nature of decision making about the purchase of Trident that largely took place outside parliamentary control.^95^ Here, MCANW echoed a contemporary political concern. Segments of the media and CND condemned the British government for focusing almost exclusively on political elites in its civil defence plans and plotting the introduction of quasi dictatorial powers to control the general population in the aftermath of a nuclear attack.^96^

By November 1986, MCANW started to assess the impact of the TNT re-launch. John Launer pointed to two major achievements: higher coherence within MCANW as a centrally ‘co-ordinated campaigning organisation’ with a diversified agenda and its capability to identify topical issues. Or, as he euphemistically put it: ‘What MCANW is saying today, Britain will be talking about tomorrow!’ Launer remained realistic though about the impact that TNT might have on British politics; he viewed MCANW and its anti-Trident campaign as facilitators who brought the issues of defence versus health expenditure to the attention of the British public.^97^

MCANW’s diversifying agenda in the TNT update epitomised the group’s continued transformation into a multi-issue campaign. If the Strategy Group had focused almost exclusively on Trident as the main focus during the preparation of the original TNT campaign, the team was now more open towards other (often IPPNW-related) matters for ‘longer term’ planning.^98^ Consequently, the Strategy Group discussed ideas for a new national campaign ahead of the TNT re-launch. While there was agreement that MCANW should continue with its recruitment of health professionals, some team members worried the group had lost its programmatic orientation, as they regarded the organisation as ‘a nuclear disarmament campaign, not a health cuts campaign’. And this demonstrated the persistence of internal struggles over MCANW’s mission, just as John Launer had appealed to members ahead of the original TNT launch in 1985 to ‘[u]se that debate to decide what … [their] group want[ed] to say …, not just argue with each other’. Eventually, consensus emerged on a new campaign on nuclear arms and health concerns in developing nations.^99^ This new campaign built on existing IPPNW contacts in developing nations and was intended to forge partnerships between MCANW branches and local health services in ‘Third World’ nations.^100^

As early as December 1985, the Strategy Group had already devised a policy paper that urged MCANW to review its general strategy and outlined three future campaigning foci: alongside government expenditure on defence versus health in Britain and developing nations, the document proposed that the group should compare the government budget allocations for arms and medical research and discuss United States President Ronald Reagan’s Strategic Defense Initiative (‘Star Wars’).^101^ These proposals led to the development of two new campaigns alongside TNT: ‘Beds not Bombs’ (BNB) and the ‘Third World Campaign’ (TWC). With its focus on ‘local experience of cuts in health services’, TNT not only served as a blue print for BNB, but the latter campaign represented, in many ways, also a continuation of TNT, albeit with a different focus. With a London launch date set for February 1987, the Strategy Group envisaged BNB to tie in with a chief recruitment drive.^102^

From January 1987, plans for TWC took shape. Through TWC, the Strategy Group intended to establish connections between the issues of development and arms reduction by pairing MCANW branches with similar groups in the ‘Third World’ and exchanging relevant data between these twinned groups.^103^ Domestic public health care provision and health concerns in developing nations also translated into MCANW’s ‘Even Before the Bomb Drops’ information pack and exhibition.^104^ At the same time, MCANW showed growing concern over human rights, especially in the wake of a military coup in Fiji and attempts by the Turkish authorities to suppress the formation of an IPPNW affiliate in that country.^105^ While these developments were powerful indicators of MCANW’s ongoing programmatic diversification, it was at IPPNW’s 1987 World Congress in Moscow, which representatives from MCANW and other national affiliates attended, that the international umbrella organisation formally recognised its (and MCANW’s) new status as a multi-issue campaign.^106^

## Conclusions

Taking place in 1985–86, when tensions between the superpowers relaxed, and combining MCANW’s original aim of nuclear disarmament with wider issues of health inequality and anti-militarism at home and in developing nations, TNT marked an important moment in the development of MCANW and medical activism more widely. Above all, it demonstrated the extension of the group’s campaigning focus to cover a broader remit both programmatically (beyond nuclear-weapons-related matters) and geographically (beyond Britain and other Western nations in the Global North). This shift was significant for MCANW and anti-nuclear politics more generally, for this strategic mobilisation of national and global health and welfare priorities made the group’s activism relevant to a larger target audience. At the same time, this new emphasis revealed similarities between MCANW and the evolution of other British NGOs from single- into multi-issue campaigns during the mid-1980s. With its anti-militarist critique of the Thatcher Government’s prioritisation of military over health spending, MCANW also took a more politicised approach to campaigning than ever before. Moreover, TNT also displayed a greater level of professionalisation of the group’s approaches and methods as well as inclusivity towards health workers, especially nurses.

While it is impossible to gauge the exact impact that TNT had on Britons’ beliefs about nuclear weapons and health inequality at home and in developing nations, some observations about the original campaign and its update, especially their arguments and rhetoric, can be made. Above all, legitimate questions remain about the feasibility of MCANW’s proposals for the cancellation of the Trident system and the subsequent re-allocation of those funds to the NHS and foreign aid budgets, as such a breach of contract would most likely result in legal charges and fines from the manufacturers of the weapon system. Perhaps, TNT’s central line should then not be taken at face value but rather as a means to raise public awareness about the cost of nuclear weapons and the state of the NHS at a time of economic and social depravation; for the welfare state came to represent such a pivotal British institution that virtually touched upon every Briton’s life after the end of the Second World War. This was also visible during Vote Leave’s campaign ahead of the 2016 referendum over Britain’s membership of the European Union (EU) where the group claimed that the sum of £350 million per week could be spent on the NHS instead of paying it to the EU, if Britain left that organisation. Shortly after the referendum, however, Vote Leave quietly abandoned this pledge: not only did the figure of £350 million not add up, but its proposal for a simple re-allocation of Britain’s EU payments to the NHS proved unfeasible.^107^

Apart from relying on the state of the NHS to illustrate health inequality, TNT had several internal and external impacts. Externally, the articulation of more pronounced political statements certainly made MCANW appear more coherent and convincing in public. Yet, unlike CND, it remained an organisation of medical activists based on its members’ ‘contributory expertise’ that avoided closer ties with groups of the anti-nuclear weapons mass movement. Internally, TNT inspired other working groups within MCANW to take more political stances.^108^ Although the campaign helped MCANW to recruit more members and increase the group’s inclusivity through greater numbers of health workers joining the organisation, there remained a sense of exclusivity of medical professionals, particularly amongst nurses organised in MCANW. Some nurses and other health workers even felt that MCANW’s power structure mirrored similar hierarchies present in the NHS.^109^

MCANW continued to embrace issues outside its traditional remit of nuclear disarmament, and, in 1992, in a most pragmatic move, the group merged with its sister organisation MAPW to form the multi-issue campaign Medical Action for Global Security (MEDACT). While the new organisation continued to push for increased government expenditure on health care worldwide (at the expense of national defence budgets) and a comprehensive nuclear test ban treaty, it had a much more diverse agenda than MCANW. Not only did MEDACT look into civilian nuclear energy and its health and environmental impacts, but it expanded its remit even beyond nuclear matters into four main areas: ‘Peace and Justice’, ‘Climate and Environment’, ‘Economic Justice’ and ‘Human Rights’.^110^

Some 30 years after the original TNT launch, at a time when the House of Commons voted in favour of the Trident replacement programme and the NHS was in a critical state, the campaign’s central anti-militarist argument about the proportionality between government spending on defence versus health care was back on the political agenda. MEDACT condemned the plans to replace Trident and participated in a large anti-Trident rally in London. At the same time, CND followed a similar anti-militarist argument as proposed by MCANW in TNT, making ‘the economic case against Trident’. This included a diversion of funds from that weapons programme to the NHS. Yet, 30 years on, TNT appeared to have been forgotten, and the campaigning by medical professionals, health workers and anti-nuclear weapons activists failed to reach the same intensity and vigour as in the mid-1980s.^111^ Nevertheless, an analysis of TNT offers crucial insight into major features of the history, nature and ideologies of medical activism concerning health inequality and anti-militarism in 1980s Britain and beyond.

## Footnotes

1 ‘Jeremy Hunt mobbed by protesters at Conservative conference in Manchester’, *BBC News* online, 4 October 2015, available at: <http://www.bbc.co.uk/news/uk-politics-34439097>, (accessed 23 February 2018).

2 John Simpson, ‘The United Kingdom and the Nuclear Future: The Strength of Continuity and the Chance for Change’, *Nonproliferation Review*, 2007, 14, 227–49, 242–3; Peter Hennessy and James Jinks, *The Silent Deep: The Royal Submarine Service since 1945* (London: Allen Lane, 2015), 469–515.

3 Christian Jenssen, ‘“Die Aufrüstung ist die Mikrobe des Krieges …”: Rudolf Virchow (1821–1902), schillernder “Apostel des Friedens und der Versöhnung”’, in Thomas M. Ruprecht and Jenssen, eds, *Äskulap oder Mars? Ärzte gegen den Krieg* (Bremen: Donat, 1991), 75–98. This article follows Cynthia Cockburn’s definition of ‘anti-militarism’ as any opposition to ‘military rule, high military expenditure or the imposition of foreign [military] bases’ in *Anti-Militarism: Political and Gender Dynamics of Peace Movements* (Basingstoke: Palgrave Macmillan, 2012), 2.

4 CND, *Who Opposes Trident?* (London: CND Publications, n.d.), unpaginated, the British Library of Political and Economic Science Archives, London School of Economics, London, United Kingdom, CND Papers, CND/2008/7/2/1.

5 Nick Lewer, *Physicians and the Peace Movement* (London: Cass, 1992), 80–2; MCANW, ‘Press Information: 23rd September 1985, Background Information on MCANW’, Medact Archive, Archive and Manuscript Collection, Wellcome Library, London, United Kingdom (hereafter SA/MED), SA/MED/F/4/4. Note that TNT was also known as ‘Trident or Health?’.

6 Eleanor Davey, ‘Famine, Aid, and Ideology: The Political Activism of Médecins sans Frontières in the 1980s’, *French Historical Studies*, 2011, 34, 529–58; Lewer, *Physicians and the Peace Movement*; Lisa Rumiel, ‘Exposing the Cold War Legacy: The Activist Work of Physicians for Social Responsibility and International Physicians for the Prevention of Nuclear War, 1986 and 1992’, in Virginia Berridge and Martin Gorsky, eds, *Environment, Health, and History* (Basingstoke: Palgrave Macmillan, 2012), 224–43; Ruprecht and Jenssen, eds, *Äskulap oder Mars?*; Cockburn, *Anti-Militarism*; Kevin Morgan, ‘Militarism and Anti-Militarism: Socialists, Communists and Conscription in France and Britain 1900–1940’, *Past and Present*, 2009, 202, 207–44; Virginia Berridge, ed., *Inequalities and Health*, spec. issue of *Contemporary British History*, 2002, 16; Paul Farmer, *Infections and Inequalities: The Modern Plagues* (Berkeley: University of California Press, 1999).

7 MCANW, ‘Press Information: Press Release Sept 23rd 1985. Health Not Trident, Treatment Not Trident’, 2, 3, SA/MED/F/4/1.

8 See Nick Crawson, Matthew Hilton and James McKay, eds, *NGOs in Contemporary Britain: Non-State Actors in Society and Politics since 1945* (Basingstoke: Palgrave Macmillan, 2009); Matthew Hilton et al., *The Politics of Expertise: How NGOs Shaped Modern Britain* (Oxford: Oxford University Press, 2013), 218–20; Rudolf Klein, *The New Politics of the NHS: From Creation to Reinvention*, 7th edn (London: Radcliffe, 2013); Rodney Lowe, *The Welfare State in Britain since 1945*, 3rd edn (Basingstoke: Palgrave Macmillan, 2005); Pat Thane, *The Foundations of the Welfare State*, 2nd edn (Harlow: Longman, 1996); Ben Jackson and Robert Saunders, eds, *Making Thatcher’s Britain* (Cambridge: Cambridge University Press, 2012); Charles Moore, *Margaret Thatcher: The Authorized Biography*, 3 vols (London: Allen Lane, 2013– ), I and II; Richard Vinen, *Thatcher’s Britain: The Politics and Social Upheaval of the Thatcher Era* (London: Pocket, 2009); Holger Nehring, *Politics of Security: British and West German Protest Movements and the Early Cold War, 1945**–**1970* (Oxford: Oxford University Press, 2013); Mark Phythian, ‘CND’s Cold War’, *Contemporary British History*, 2001, 15, 133–56; Richard Taylor, *Against the Bomb: The British Peace Movement, 1958**–**1965* (Oxford: Clarendon Press, 1988).

9 MCANW, ‘Constitution’, July 1983, The Craig Medical Peace Movements Archive, J. B. Priestley Library Special Collections, University of Bradford, Bradford, United Kingdom (hereafter CMPMA), 3/3; Lewer, *Physicians and the Peace Movement*, 81–2.

10 Harry Collins and Robert Evans, *Rethinking Expertise* (Chicago: University of Chicago Press, 2014), 14; Steven Epstein, *AIDS, Activism, and the Politics of Knowledge* (Berkeley: University of California Press, 1996), 8.

11 MCANW, ‘Press Information: 23rd September 1985, Background Information on MCANW’; Virginia Berridge and Alex Mold, ‘Professionalisation, New Social Movements and Voluntary Action in the 1960s and 1970s’, in Matthew Hilton and James McKay, eds, *The Ages of Voluntarism: How We Got to the Big Society* (Oxford: Oxford University Press, 2011), 114–34, 115–16.

12 Lawrence S. Wittner, *The Struggle against the Bomb*, 3 vols (Stanford, CA: Stanford University Press, 1993–2003), *III*, 229; MCANW, ‘Breakdown of MCANW Membership on a Total of 3369—14th March 1986 (excluding Scotland)’, 24 March 1986, SA/MED/C/2/2/4.

13 ‘MCANW Executive Meeting. Thursday 10 June 1982’, 2–3, SA/MED/C/2/1/1; Elaine Cundy, ‘MCANW Nurses Working Party’, n.d., CMPMA, 3/4.

14 Stephen Brooke, ‘The Sphere of Sexual Politics: The Abortion Law Reform Association, 1930s to 1960s’, in Crawson, Hilton and McKay, eds, *NGOs in Contemporary Britain*, 77–94; Diarmaid Kelliher, ‘Solidarity and Sexuality: Lesbians and Gays Support the Miners 1984–5’, *History Workshop Journal*, 2014, 77, 240–62; Christopher Moores, ‘The Progressive Professionals: The National Council for Civil Liberties and the Politics of Activism in the 1960s’, *Twentieth Century British History*, 2009, 20, 538–60, 539; John Stewart, *‘The Battle for Health’: A Political History of the Socialist Medical Association, 1930**–**1951* (Aldershot: Ashgate, 1999); Steve Watkins, *Medicine and Labour: The Politics of a Profession* (London: Lawrence and Wishart, 1987), 61–5.

15 ‘MCANW National Council Meeting. Sunday 25 October 1981’, 4, SA/MED/C/1/1/1; ‘MCANW Executive Meeting. Thursday 10 June 1982’, 2; ‘No. 8. Minutes. Executive Committee Meeting, Thursday 2nd June 1983’, 2, all in SA/MED/C/2/1/1; Watkins, *Medicine and Labour*, 20–1, 69, 183.

16 Alex Poteliakoff and Patricia Craig, ‘Four Decades of the Medical Association for Prevention of War’, *Medicine and War*, 1991, 7, 173–84, 176; ‘International Physicians for the Prevention of Nuclear War’, *The Lancet*, 1985, 326, 1289.

17 Bernard Lown and John O. Pastore, ‘A Medical Prescription for Survival’, *The Lancet*, 1985, *326*, 1285–6; Barry S. Levy and Victor W. Sidel, ‘War and Public Health: An Overview’, in Levy and Sidel, eds, *War and Public Health*, 2nd edn (Oxford: Oxford University Press, 2008), 3–20, 15–19; Lewer, *Physicians and the Peace Movement*, 45–8; N. Arthur Coulter, ‘Militarism: A Psychosocial Disease’, *Medicine and War*, 1992, 8, 7–17, 12, 14. ‘Militarism’ is defined here, according to Martin Shaw, as ‘*the penetration of social relations in general by military relations* [orig. emphasis]’ in ‘Twenty-First Century Militarism: A Historical-Sociological Framework’, in Anna Stavrianakis and Jan Selby, eds, *Militarism and International Relations: Political Economy, Security, Theory* (London: Routledge, 2013), 19–32, 20.

18 David Edgerton, ‘Liberal Militarism and the British State’, *New Left Review*, 1991, 185, 138–69, 164–6; David Edgerton, *Warfare State: Britain, 1920**–**1970* (Cambridge: Cambridge University Press, 2006).

19 G. H. Adam et al., ‘The War Mentality: An Address to Statesmen’, *The Lancet*, 1935, 226, 907; Craig and Jenssen, ‘Zwischen Utopie, hilfloser Analyse und Kriegsvorbereitung’, 310–13; Lewer, *Physicians and the Peace Movement*, 52–7; Peter van den Dungen, ‘Dr. Emil Flusser: Forgotten Precursor of the Medical Peace Movement’, *Medicine, Conflict and Survival*, 1996, 12, 90–106, 90, 91, 94.

20 ‘MCANW Strategy Group: First Report to National Council (September 14th 1985)’, 1, CMPMA, 3/13; MCANW, ‘Press Information: Sep 24.85, Report on Launch of Campaign Treatment Not Trident’, SA/MED/F/4/4. Black faced some criticism over his support of TNT, ‘Black’s Accident Spotting’, *Guardian*, 24 September 1985, 32.

21 MCANW, ‘Press Information: “Treatment Not Trident.” Campaign Activities’, n.d., SA/MED/F/4/1.

22 MCANW, ‘Press Information: Treatment Not Trident’, n.d., attached to letter, Bernadette Hayes to MCANW branch co-ordinators, 9 October 1985, Archive of the Medical Association for the Prevention of War, J. B. Priestley Library Special Collections, University of Bradford, Bradford, United Kingdom (hereafter MAPW Archive), M. 2; Martin Ceadel, *Thinking about Peace and War* (Oxford: Oxford University Press, 1987), 90–1.

23 MCANW, *Trident: A Tragedy for the Health Services* (n.p.: MCANW, 1985), 2, 7–9, SA/MED/F/4/4; MCANW, ‘Press Information: 23rd September 1985, Background Information on MCANW’; Will Howard, ‘Memorandum: Key Points in Developing the Freeze Dialogue’, 13 April 1985, SA/MED/J/1/6; Bradford Martin, *The Other Eighties: A Secret History of America in the Age of Reagan* (New York: Hill and Wang, 2011), 3–24.

24 Len Scott, ‘Labour and the Bomb: The First 80 Years’, *International Affairs*, 2006, 82, 685–700, 691; Richard Taylor, ‘The Labour Party and CND’, in Richard Taylor and Nigel Young, eds, *Campaigns for Peace: British Peace Movements in the Twentieth Century* (Manchester: Manchester University Press, 1987), 100–30, 120–3.

25 Official Group on Nuclear Weapons and Public Opinion, ‘An Assessment of the Public Nuclear Debate, D/DS17/2/16/15’, 18 December 1984, 8, the National Archives, Kew, Richmond, UK (hereafter TNA), FCO 46/4188.

26 Carver, cited in MCANW, *Trident or Health?* (London and Cambridge: MCANW, [1985]),1, SA/MED/F/4/2.

27 See, for example, ‘MCANW: Policy Making Conference, 13 & 14 November 1982’; MCANW, ‘Resolutions from the Policy Conference held in London on November 13th/14th, 1982’, 23 November 1982, 2, attached to letter, P. G. Mann to B. A. Staines, 14 December 1982, all in CMPMA, 3/2; MCANW, ‘Petition to Her Majesty’s Government to Reverse the Decision to Introduce the Trident Weapon System’, 24 September 1984, MAPW Archive, M. 2; ‘MCANW Chair Leads Deputation to Number 10’, *MCANW National Newsletter*, 1985, 13, 7, CMPMA, 3/12.

28 ‘Annual General Meeting. MCANW. Minutes: AGM 25th June 1983’, in ‘Interim Report: Summer 1983’, 6–7, SA/MED/C/1/1/1. Moore, *Margaret Thatcher*, *II*, 50; David Josephs, ‘1983 to 1988—The BMA and the Nuclear Issue’, *Medicine and War*, 1989, 5, 139–40; Watkins, *Medicine and Labour*, 183–8.

29 ‘From the ARM: Money, Manpower, and Management’, *British Medical Journal*, 1984, 289, 199–203; David Hencke, ‘BMA Says Arms Cash Cuts Could Pay for Health Care’, *Guardian*, 6 July 1984, 3; Nicholas Timmins, ‘British Medical Association’, *The Times*, 6 July 1984, 3; Joanna Francis, ‘Doctors Vote for Arms Cut’, *Sanity*, 1984, 8, 6; Watkins, *Medicine and Labour*, 150.

30 MCANW, *Trident or Health?*,1.

31 MCANW, ‘Press Information: 23rd September 1985, Background Information on MCANW’; MCANW, *Trident: A Tragedy for the Health Services*, 2, 7–9.

32 MCANW, ‘Press Information: Press Release Sept 23rd 1985. Health Not Trident, Treatment Not Trident’; Hilton et al., *The Politics of Expertise*, 127–31. According to the Parliamentary Archives, an early day motion was neither tabled during the 1985–86 nor the early 1986–87 sessions; Richard Ward to author, email, 15 April 2016.

33 ‘MCANW Executive Meeting. Thursday 10 June 1982’, 2; ‘Minutes No. 20. MCANW Executive Committee Meeting held on Saturday 15th December 1984’, 2, all in SA/MED/C/2/1/1; ‘Minutes No. 12. MCANW National Council Meeting held at Friends House, Euston Road, London, on Saturday, 16th March 1985’, 2, SA/MED/C/1/1/1.

34 Becker-Schaum et al., ‘Introduction: The Nuclear Crisis, NATO’s Double-Track Decision, and the Peace Movement of the 1980s’ in Becker-Schaum et al., eds, *The Nuclear Crisis: The Arms Race, Cold War Anxiety, and the German Peace Movement of the 1980s*, New York: Berghahn Books, 2016, 1–36, 23–25.

35 Hilton et al., *The Politics of Expertise*, 62–3, 228.

36 Christoph Laucht, ‘Scientists, the Public, the State, and the Debate over the Environmental and Human Health Effects of Nuclear Testing in Britain, 1950–1958’, *Historical Journal*, 2016, 59, 221–51.

37 Hilton et al., *The Politics of Expertise*, 218–20.

38 Black and Farrow cited in Andrew Veitch, ‘Doctors Prescribe Cure for Trident Spending’, *Guardian*, 24 September 1985, 3; ‘MCANW: Expenditure on Armaments and on Health Care’, *The Lancet*, 1985, 326, 735; Su Maddock, ‘Treatment Not Trident’, *MCANW National Newsletter*, 1985, 15, 1–2, 2, CMPMA, 3/12; MCANW, ‘Press Information: 23rd September 1985, Background Information on MCANW’; and press clippings, all in SA/MED/F/4/4.

39 MCANW, ‘Press Information: Sep 24.85, Report on Launch of Campaign Treatment Not Trident’.

40 ‘Trident: £7,260 Million Worth of Death’, *Sanity*, 1979, 6, 9; ‘How Much Will Trident Cost?’, *The Times*, 16 July 1980, 17; Judith Reppy and Harry Dean, ‘Britain Buys the Trident’, *Bulletin of the Atomic Scientists*, 1980, 36, 26–31; ‘Defence Debate’, *News at Ten* (ITV Late Evening News), 7 July 1981; Lawrence Freedman, ‘Britain: The First Ex-Nuclear Power?’, *International Security*, 1981, 6, 80–104.

41 John Baylis and Kristan Stoddart, *The British Nuclear Experience: The Role of Beliefs, Culture, and Identity* (Oxford: Oxford University Press, 2015), 151–69; Andrew Dorman, ‘John Nott and the Royal Navy: The 1981 Defence Review Revisited’, *Contemporary British History*, 2001, 15, 98–120, 118; Moore, *Margaret Thatcher*, I, 571–3; ‘Counting the Warheads’, *Guardian*, 6 October 1981, 12.

42 MCANW, *Trident: A Tragedy for the Health Services*, 7; MCANW, ‘Press Information: Sep 24.85’; ‘Trident Costs’, *MCANW National Newsletter*, 1985, 13, 7, CMPMA, 3/12.

43 Keith Hartley, ‘The Economics of UK Nuclear Weapons Policy’, *International Affairs*, 2006, 82, 675–-84, 678–9; Mort, *Building the Trident Network*, 187 n 5; Margaret Thatcher, *The Downing Street Years* (London: Harper, 1993), 244, 248; Moore, *Margaret Thatcher*, I, 572–3.

44 MCANW, *Trident: A Tragedy for the Health Services*, 1.

45 Arnold S. Relman, ‘The New Medical-Industrial Complex’, *New England Journal of Medicine*, 1980, 303, 963–70.

46 ‘IPPNW: 4th Congress Meets in Helsinki’, *The Lancet*, 1984, 323, 1363–4, 1363; MCANW, *Trident: A Tragedy for the Health Services*, 1, 6.

47 Steffie Woolhandler and David U. Himmelstein, ‘Militarism and Mortality: An International Analysis of Arms Spending and Infant Death Rates’, *The Lancet*, 1985, 325, 1375–8; Peter H. Whincup, letter to the editor, *The Lancet*, 1985, 326, 46; Douglas Holdstock, ‘Health and the Arms Race’, *Medicine and War*, 1985, 1, 83–5. For a contemporary critique of this correlation, see Petr Skrabanek, letter to the editor, *The Lancet*, 1985, 326, 46.

48 Charles Webster, ‘Investigating Inequalities in Health before Black’, *Contemporary British History*, 2002, 16, 81–104; Peter Townsend and Nick Davidson, eds, *Inequalities in Health: The Black Report* (Harmondsworth: Penguin, 1982); Mel Bartley, *Health Inequality: An Introduction to Theories, Concepts and Methods* (Cambridge: Polity Press, 2004), 1; MCANW, *Trident: A Tragedy for the Health Services*, 6; MCANW, *Trident or Health? Treatment Not Trident Campaign Update. 23 September 1986* (London: MCANW, 1986), 4, SA/MED/F/4/6.

49 Townsend and Davidson, eds, *Inequalities in Health*; Peter Townsend, Margaret Whitehead and Nick Davidson, ‘Introduction to *Inequalities in Health*, 1992 Edition’, in Douglas Black et al., *Inequalities in Health: The Black Report and The Health Divide* (London: Penguin, 1992), 1–27, 2–3.

50 Sally Macintyre, ‘The Black Report and Beyond: What Are the Issues?’, *Social Science and Medicine*, 1996, 44, 723–45, 723; Christopher Hamlin, *Public Health and Social Justice in the Age of Chadwick: Britain, 1800**–**1854* (Cambridge: Cambridge University Press, 1998).

51 Patrick Jenkin, ‘Foreword’, in Townsend and Davidson, eds, *Inequalities in Health*, unpaginated; Townsend, Whitehead and Davidson, ‘Introduction to *Inequalities in Health*, 1992 Edition’, 5; MCANW, *Trident: A Tragedy for the Health Services*, 6; Minerva, ‘Views’, *British Medical Journal*, 1980, 281, 690; ‘Inequalities in Health’, *British Medical Journal*, 1980, 281, 691; ‘Equalities and Inequalities in Health’, *British Medical Journal*, 1980, 281, 762–3; ‘An Endangered Species’, *British Medical Journal*, 1980, 281, 1662–3. The methodologies used in the Black Report were not undisputed: ‘Inequalities in Health’, *The Lancet*, 1980, 316, 513.

52 Working Group on Inequalities in Health, ‘Preface’, in Townsend and Davidson, eds, *Inequalities in Health*, unpaginated; Rodney Deitch, ‘Unwanted Legacy from Jenkin’s Predecessor’, *The Lancet*, 1980, 316, 545.

53 ‘Exchange of Letters’, *The Times*, 16 July 1980, 6.

54 John Appleby, ‘Government Funding of the UK National Health Service: What Does the Historical Record Reveal?’, *Journal of Health Services Research and Policy*, 1999, 4, 79–89, 79, 83; Martin Gorsky, ‘The British National Health Service 1948–2008: A Review of the Historiography’, *Social History of Medicine*, 2008, 21, 437–60, 445–6; Ben Jackson and Robert Saunders, ‘Introduction: Varieties of Thatcherism’, in Jackson and Saunders, eds, *Making Thatcher’s Britain*, 1–21, 15; Lowe, *The Welfare State in Britain since 1945*, 340–75; Nicholas Timmins, *The Five Giants: A Biography of the Welfare State*, rev. edn (London: HarperCollins, 2001), 369–494.

55 Simon Szreter, *Health and Wealth: Studies in History and Policy* (Rochester: University of Rochester Press, 2005), 14–15.

56 Stephen Brooke, ‘Living in “New Times”: Historicizing 1980s Britain’, *History Compass*, 2014, 12, 20–32, 27.

57 MCANW, *Trident: A Tragedy for the Health Services*, 1–2.

58
*Ibid*., 2–3; Radical Statistics Health Group, ‘Unsafe in Their Hands: Health Service Statistics for England’, *Radical Statistics*, 1985, 33, 4–14; Radical Statistics Nuclear Disarmament Group, *The Nuclear Numbers Game: Understanding the Statistics behind the Bombs* (London: Radical Statistics Group, 1982), 95.

59 MCANW, *Trident: A Tragedy for the Health Services*, 4–6.

60 Peter Broderick to F. H. Moberley, 23 December 1981, TNA, FCO 46/2696.

61 ‘IPPNW: 4th Congress Meets in Helsinki’, 1363.

62 Andrew Jones, ‘Band Aid Revisited: Humanitarianism, Consumption and Philanthropy in the 1980s’, *Contemporary British History*, 2017, 31, 189–209, 192; Jones, ‘The Disasters Emergency Committee (DEC) and the Humanitarian Industry in Britain, 1963–85’, *Twentieth Century British History*, 2015, 26, 573–601; Davey, ‘Famine, Aid, and Ideology’.

63 ‘Trident or Health: World Health Needs’, n.d., unpaginated, SA/MED/F/4/2.

64
*Ibid*.; Hilton et al., *The Politics of Expertise*, 235; MAPW, ‘Appeal for Phased World Disarmament, Both Nuclear and Conventional’, *The Lancet*, 1980, 316, 739.

65 Willy Brandt et al., *North**–**South: A Programme for Survival. Report of the International Commission on International Development Issues* (London: Pan, 1980); Lewer, *Physicians and the Peace Movement*, 82; Peter Merseburger, *Willy Brandt, 1913**–**1992: Visionär und Realist* (Stuttgart: Deutsche Verlags-Anstalt, 2002), 739–40.

66 ‘Trident or Health: World Health Needs’.

67 Berridge and Mold, ‘Professionalisation, New Social Movements and Voluntary Action in the 1960s and 1970s’, 114–34; Jones, ‘The Disasters Emergency Committee (DEC) and the Humanitarian Industry in Britain’, 573–601.

68 ‘Minutes MCANW Executive Committee Meeting. Thursday 26 November 1981’, SA/MED/C/2/1/1.

69 ‘MCANW Strategy Group: First Report to National Council (September 14th 1985)’, 1; ‘Minutes No. 12. MCANW National Council Meeting held at Friends House, Euston Road, London, on Saturday, 16th March 1985’, 2, SA/MED/C/1/1/1; ‘Minutes No. 20. MCANW Executive Committee Meeting held on Saturday 15th December 1984’, 2; ‘Minutes No. 22. MCANW Executive Committee Meeting held on Saturday 30th March 1985’, 2, all in SA/MED/C/2/1/1.

70 ‘MCANW Strategy Group: First Report to National Council (September 14th 1985)’, 1; John Launer to MCANW branch coordinators, n.d., 1, SA/MED/F/4/5.

71 ‘Trident’, ‘Barrow Day of Action Against Trident’, ‘Trident Costs’, *MCANW National Newsletter*, 1985, 13, 6, 7, CMPMA, 3/12; ‘No. 21. Minutes of MCANW Executive Committee Meeting held on Saturday 9th February 1985’, 3; ‘Minutes No. 23. MCANW Executive Committee Meeting held on Saturday 11th May 1985’, 2, both in SA/MED/C/2/1/1; ‘Minutes: MCANW National Council Meeting. Saturday 8 May 1982’, 4, SA/MED/C/1/1/1; ‘Defence Estimates’, House of Commons Debate, 13 June 1985, Hansard, 80, 1035-66, 1068–115; Strategy Committee to [branch] coordinators and parliamentary monitors, 14 May 1985, 1–2, SA/MED/F/4/4.

72 ‘Trident or Health’, n.d.; ‘Trident or Health: Lobbying Your MP—Local Constituency Work’, n.d.; ‘Trident or Health: World Health Needs’, 1; Su Maddock, ‘Trident Or Health: Campaigning Notes’, June 1985, 1, all in SA/MED/F/4/4.

73 John Launer to branch coordinators, 3 July 1985; John Launer to branch coordinators, 28 August 1985, 1, all in SA/MED/F/4/1; ‘Contents of Briefing Pack for National Campaign on Trident versus Health’, June 1985, SA/MED/F/7/1; ‘Press Coverage for 1985 on Health Service’, MCANW, ‘Press Information: For Branch Press Officers. Press Coverage for 1985 on Health Service’, n.d.; York and Humberside MCANW, ‘Trident Or Treatment?’, n.d., all in SA/MED/F/4/4.

74 ‘Minutes No. 25. MCANW Executive Committee Meeting held on Saturday 5th October 1985’, 2, SA/MED/C/2/1/1.

75 ‘Treatment or Health?’, *British Medical Journal*, 1985, 291, 976; Su Maddock, ‘Press Information: Report on the Trident or Health Campaign, “Treatment Not Trident”’, 17 November 1985, SA/MED/F/4/1.

76 ‘Minutes No. 25. MCANW Executive Committee Meeting held on Saturday 5th October 1985’, 1; ‘Minutes No. 26. MCANW Executive Committee Meeting held on Saturday 23rd November 1985’, 1, all in SA/MED/C/2/1/1.

77 J. H. Marks to Sue Maddock, 14 August 1985; John Stevens to Sue Maddock, 14 August 1985; Canon Paul Oestreicher to Stephen Farrow, 3 September 1985; Bath Anti-Trident Action Group to MCANW, 11 September 1985, 1, all in SA/MED/F/4/1.

78 Su Maddock to branch coordinators and strategy links, 12 November 1985, SA/MED/F/4/1; Jane Dibblin, ‘Anti-Nuclear Nurses’, *Sanity*,1986, 8, 44–5, 45.

79 Maddock, ‘Press Information’; Maddock to branch press officers, 27 February 1986, SA/MED/F/4/4; ‘No. 16. Minutes of MCANW National Council held on Saturday 21st June 1986’, 4, SA/MED/C/1/1/1.

80 Gillian Reeve, ‘Press Information: Medical Campaign Against Nuclear Weapons. Not for Release before 0001 23 September 1986, Treatment Not Trident—One Year Later’, n.d., 1, SA/MED/F/4/2.

81 ‘Minutes No. 26. MCANW Executive Committee Meeting held on Saturday 23rd November 1985’, 2; Su Maddock to branch press officers, 27 February 1986, SA/MED/F/4/4; Reeve, ‘Press Information’, 1.

82 Strategy Group to all strategy and branch coordinators, January 1986, SA/MED/C/2/2/2; ‘No. 27. Minutes of Executive Committee Meeting held on Saturday 11th January 1986’, 2; ‘Minutes No. 28. MCANW Executive Committee Meeting held on Saturday 15th March 1986’, 1, all in SA/MED/C/2/1/1; ‘Minutes. No. 15. MCANW National Council Meeting held on Saturday 15th February 1986’, 2, SA/MED/C/1/1/1; ‘Treatment Not Trident’, *MCANW National Newsletter*, 1986, 16, 4, CMPMA, 3/15; MCANW, ‘Why Are We Waiting?’, *Sanity*, 1986, 6, 47; Reeve, ‘Press Information’, 1; ‘“For the Cost of One Trident” Billboards’, *MCANW National Newsletter*, 1986, 17, 3, CMPMA, 3/12; John Launer, ‘Personal View’, *British Medical Journal*, 1986, 292, 407; ‘Annual General Meeting & Conference. 19th/20th April 1986, London’, 1, SA/MED/K/1/4.

83 ‘MCANW. Strategy Group. Minutes of the Meeting held on 6 September 1986’, 1; Carol-Ann Allen to MCANW members, 17 September 1986, all in SA/MED/F/1/3.

84 Reeve, ‘Press Information’, 1; ‘One Year of “Trident or Health?”’, *British Medical Journal*, 1986, 293, 825.

85 Reeve, ‘Press Information’, 2.

86
*Ibid*., 2–3. MCANW, *Trident or Health? Treatment not Trident Campaign Update*, 1, 2, 10–11, 13, 15; Vinen, *Thatcher’s Britain*, 125.

87 E. W. Bakke, *The Unemployed Man: A Social Study* (London: Nisbet, 1933); G. D. H. Cole and M. I. Cole, *The Condition of Britain* (London: Gollancz, 1937); P. Eisenberg and P. F. Lazarsfeld, ‘The Psychological Effects of Unemployment’, *Psychological Bulletin*, 1938, 35, 358–90.

88 ‘No. 16. Minutes of MCANW National Council held on Saturday 21st June 1986’, 2, SA/MED/C/1/1/1. A notable exception is Martin Dace, *Everything You Wanted to Know about Radiation and Health (But Couldn’t Find Anyone to Ask)* (London: MCANW, 1987), SA/MED/K/1/3.

89 MCANW, ‘Trident or Health? Treatment Not Trident Campaign. Embargoed Until: 23 September 1986’, n.d., 2, SA/MED/F/4/2.

90 Hilton et al., *The Politics of Expertise*, 219; Lawyers for Nuclear Disarmament, *Pamphlet One: The Illegality of Nuclear Warfare* (London: Lawyers for Nuclear Disarmament, 1982), SA/MED/J/1/12. Richard Maguire historicises this argument in ‘The Use of Nuclear Weapons: Mass Killing and the United Kingdom Government’s Nuclear Weapons Programme’, *Journal of Genocide Research*, 2007, 9, 389–410.

91 MCANW, *Trident or Health? Treatment not Trident Campaign Update*, 22.

92
*Ibid*.; Reeve, ‘Press Information’, 1.

93 MCANW, *Trident or Health? Treatment not Trident Campaign Update*, 17–18; MCANW, *Trident: A Tragedy for the Health Services*.

94 MCANW, *Trident or Health? Treatment not Trident Campaign Update*, 18–20; Edgerton, ‘Liberal Militarism and the British State’, 164–6; Maggie Mort, *Building the Trident Network: A Study of the Enrollment of People, Knowledge, and Machines* (Cambridge, MA: MIT Press, 2002), 79–108, 138–41; ‘Don’t Forget Trident—It’s Economic Ruin for Us’, *Sanity*, 1982, 5, 17; Alan Millburn, ‘Work for the Future: Barrow, Trident and the Alternative’, *Sanity*, 1984, 1, 17–21; Brian P. Jamieson, ‘Britain’s National Deterrent: Scotland’s Answer to the Cycle of Unemployment?’, *Contemporary British History*, 2007, 21, 449–69.

95 MCANW, *Trident or Health? Treatment not Trident Campaign Update*, 21.

96 Central Office of Information, *Protect and Survive* (London: HMSO, 1980); E. P. Thompson, *Protest and Survive* (London: CND; Nottingham: Bertrand Russell Peace Foundation, 1980); Philip Bolsover, *Civil Defence: The Cruellest Confidence Trick* (London: CND, 1982); Duncan Campbell, *War Plan UK: The Truth about Civil Defence in Britain* (London: Burnett, 1982), 177–284; James Stafford, ‘“Stay at Home”: The Politics of Nuclear Civil Defence, 1968–83’, *Twentieth Century British History*, 2012, 23, 383–407.

97 ‘MCANW. Strategy Group. Minutes of Meeting held on 1 November 1986’, SA/MED/F/1/3; John Launer, ‘Trident or Health?’, *MCANW National Newsletter*, 1986, 17, 1, CMPMA, 3/12.

98 ‘MCANW Strategy Group: First Report to National Council (September 14th 1985)’, 1; ‘Minutes No. 13 MCANW National Council Meeting held on Saturday 14th September 1985’, 2, SA/MED/C/1/1/1.

99 ‘MCANW. Strategy Group. Minutes of the Meeting held on 6 September 1986’, 1–2, SA/MED/F/1/3; John Launer to branch coordinators, 3 July 1985, 2, SA/MED/F/4/1.

100 ‘MCANW. Strategy Group. Minutes of the Meeting held on 6 September 1986’, 2.

101 ‘Minutes No. 14. MCANW National Council Meeting held on Saturday 7th December 1985’, 2, SA/MED/C/1/1/1.

102 MCANW National Council. No. 17. Minutes of the NC Meeting held on Saturday 11 October 1986’, 2, SA/MED/C/1/1/1; ‘MCANW. Strategy Group. Minutes of a Meeting held on 6 December 1986’, 1, SA/MED/C/2/2/2.

103 ‘MCANW. Minutes of the Strategy Group Meeting, 9 January 1987’; ‘MCANW. Minutes of the Strategy Group Meeting, 7 February 1987’, 1, attached to letter, Carol-Ann Allen to Strategy Group members, 12 February 1987; ‘Strategy Group Meeting, 13 June 1987. Minutes’, 1, all in SA/MED/F/1/3; MCANW National Council. No. 17. Minutes of the NC Meeting held on Saturday 11 October 1986’.

104 Carol-Ann Allen, ‘MCANW Exhibition’, 26 October 1987; Gillian Reeve to Joan Anderson, 26 May 1988; MCANW, *Even Before the Bomb Drops: The Present Effects of Nuclear Weapons. Draft Copy* (London: MCANW, n.d.), all in SA/MED/F/8/3.

105 John Humphrey to Timori Bavadra, 10 January 1987; Mehmet Ali Dikerdem to Manda Gifford, 7 February 1987; Gifford to Dikerdem, 14 February 1987; Dikerdem to Mercy Heatly, 2 March 1987; Dikerdem to Heatly, 2 May 1987; Özen Aşut to Heatly, 13 May 1987; Heatly to Marlene Läubli, 24 June 1987; Hans Lavender to Läubli, 8 July 1987, all in CMPMA, 3/7.

106 Carol-Ann Allen to Strategy Group members, 17 June 1987, SA/MED/F/1/4; Jim Dyer, ‘International Physicians for the Prevention of Nuclear War: A New Manner of Thinking’, *The Lancet*, 1987, 329, 1383–4; ‘The Future of IPPNW’, *The Lancet*, 1987, 329, 1353–4.

107 Toby Helm, ‘Brexit Camp Abandons £350m-a-Week NHS Funding Pledge’, *Guardian*, 10 September 2016, <https://www.theguardian.com/politics/2016/sep/10/brexit-camp-abandons-350-million-pound-nhs-pledge>, (accessed 23 February 2018).

108 ‘BMA Working Party’, *MCANW National Newsletter*, 1985, 15, 5, CMPMA, 3/12.

109 Dibblin, ‘Anti-Nuclear Nurses’, 44.

110 Shirley E. Freeman, ‘Medical Action for Global Security (MEDACT): Founding Statement’, *Medicine and War*, 1992, *8*, 131-32; ‘Our Work’, https://www.medact.org/our-work/, (accessed 23 February 2018).

111 Rowena Mason, ‘May: I would authorize a nuclear strike by UK’, *Guardian*, 19 July 2016, 1, 6; Deborah Haynes, ‘I’d press the nuclear button, vows PM as she wins vote for deterrent’, *The Times*, 19 July 2016, 10–11; Medact, ‘The UK Trident Missile System Factsheet’, <http://www.medact.org/wp-content/uploads/2014/01/THE-UK-TRIDENT-MISSILE-SYSTEM-FINAL.pdf>, (accessed 23 February 2018); Rhianna Louise, ‘Our response to Trident renewal’, 21 July 2016, <https://www.medact.org/2016/blogs/response-trident-renewal/>, (accessed 23 February 2018); CND, *People not Trident: The Economic Case against Trident Replacement* (London: CND, 2015); ‘Medact at the #StopTrident March’, 28 February 2016, <https://www.medact.org/2016/actions/medact-at-the-stoptrident-march/>, (accessed 23 February 2018).

## Notes

Dr Christoph Laucht, FRHistS, FHEA, is Senior Lecturer in Modern History at Swansea University, Wales. He works on the cultural history of the nuclear age and the transnational history of the Cold War and is the author of *Elemental Germans: Klaus Fuchs, Rudolf Peierls and the Making of British Nuclear Culture, 1939**–**59* (Basingstoke: Palgrave Macmillan, 2012) and co-editor (with Tobias Hochscherf and Andrew Plowman) of *Divided, But Not Disconnected: German Experiences of the Cold War* (New York: Berghahn Books, 2010) and (with Jonathan Hogg) of *British Nuclear Culture*, spec. issue of the *British Journal for the History of Science*, 45.4 (2012).

